# Basic mechanisms for recognition and transport of synaptic cargos

**DOI:** 10.1186/1756-6606-2-25

**Published:** 2009-08-04

**Authors:** Max A Schlager, Casper C Hoogenraad

**Affiliations:** 1 Department of Neuroscience, Erasmus Medical Center, 3015GE, Rotterdam, The Netherlands

## Abstract

Synaptic cargo trafficking is essential for synapse formation, function and plasticity. In order to transport synaptic cargo, such as synaptic vesicle precursors, mitochondria, neurotransmitter receptors and signaling proteins to their site of action, neurons make use of molecular motor proteins. These motors operate on the microtubule and actin cytoskeleton and are highly regulated so that different cargos can be transported to distinct synaptic specializations at both pre- and post-synaptic sites. How synaptic cargos achieve specificity, directionality and timing of transport is a developing area of investigation. Recent studies demonstrate that the docking of motors to their cargos is a key control point. Moreover, precise spatial and temporal regulation of motor-cargo interactions is important for transport specificity and cargo recruitment. Local signaling pathways - Ca2+ influx, CaMKII signaling and Rab GTPase activity - regulate motor activity and cargo release at synaptic locations. We discuss here how different motors recognize their synaptic cargo and how motor-cargo interactions are regulated by neuronal activity.

## Introduction

Neurons are highly polarized cells with distinct membrane domains, including a single extended axon and multiple dendritic processes which contain thousands of individual synapses. Synapses are composed of a presynaptic terminal, a synaptic cleft, and a postsynaptic specialization and are the structures through which neurons communicate [1,2]. Presynaptic boutons transmit signals by releasing neurotransmitters from synaptic vesicles and postsynaptic sites receive information using neurotransmitter receptors. Most excitatory glutamate receptors, such as N-methyl D-aspartate (NMDA) receptors and α-amino-3-hydroxy-5-methyl-4-isoxazole propionate (AMPA) receptors are located at dendritic spines, whereas inhibitory glycine receptors (GlyRs) and γ-aminobutyric acid type A receptors (GABAARs) are mainly located at the shaft of dendrites. Recent studies have identified the molecular components of synapses, particularly by using proteomic strategies and have revealed that the specification of synaptic function, e.g. excitatory or inhibitory, at both pre- and post-synapses is achieved via the recruitment and assembly of distinct receptors and their associated proteins [1,3-6]. The assembly requires selective localization or stabilization of receptors at synaptic membranes [7], as well as the highly coordinated intracellular transport of synapse-associated proteins [8]. In *Drosophila*, selective synaptic cargo transport has been shown to be essential for synapse formation at the neuromuscular junction while axon outgrowth and guidance were unaffected [9].

The most widely used mechanism for intracellular trafficking involves molecular motor proteins that carry cargo directionally along a cytoskeletal track - myosins along actin and kinesins and dyneins along microtubules. Various organelles and membranous structures have been shown to move bidirectionally along the cytoskeletal network - some cargos can even switch between actin and microtubules [10]. Despite extensive knowledge on the complete genomic inventories of molecular motors in several diverse organisms [11,12] and significant progress in our understanding of neuronal transport in general [13], the cellular mechanisms responsible for synaptic cargo trafficking remain unclear. It is not fully understood how a limited number of motor proteins carries the wide variety of synaptic cargos, such as neurotransmitter receptors, ion channels, integral membrane proteins, signaling complexes, mRNAs, synaptic vesicle precursors (SVPs), Piccolo-Bassoon transport vesicles (PTVs), mitochondria or other organelles (Table 1). Emerging evidence suggests that the docking of motors to their cargos via adaptor molecules is an important mechanism to achieve transport specificity. Specific transport mechanisms are crucial for neuronal development and plasticity, such as synaptogenesis [5,9] and synaptic plasticity [14,15]. Moreover, alterations in the trafficking of synaptic receptors and associated proteins may contribute to pathologic changes in neurological and psychiatric disease [15-17].

**Table 1:** Motors and adaptors for transport of synaptic cargos

**Cargo**	**Adaptor**	**Motor**	**Reference**
**Receptor**			
NMDA Receptor (NR2B)	CASK/MALS/Mint	KIF17	[89]
AMPA Receptor (GluR1/GluA1)	SAP-97	Myosin VI	[130]
		Myosin V	[46]
AMPA Receptor (GluR2/GluA2)	GRIP1	KIF5	[88]
Glycine Receptor (GlyR)	Gephyrin/DLC	Dynein	[131]
Reelin Receptor (APOER2)	JIP	KIF5	[80]
Trk Receptor (TrkB)	Rab27B/Slp1/CRMP-2	KIF5	[127]
	DLC	Dynein	[73]
	GIPC1	Myosin VI	[133]
			
**Synaptic Vesicle Precursor (SVP)**			
VAMP2/SYP		Myosin V	[134]
Rab3	DENN/MADD	KIF1A/KIF1Bβ	[104]
PIP2		KIF1A	[66]
unknown	Liprin-α	KIF1A	[91]
			
**Piccolo-Bassoon Transport Vesicle (PTV)**			
Bassoon	DLC	Dynein	[135]
Syntaxin-1	Syntabulin	KIF5	[136]
SNAP-23/25		KIF5	[137]
			
**Mitochondria**			
Miro	Milton	KIF5	[83]
unknown	JIP	KIF5	[138]
unknown	Syntabulin	KIF5	[139]
unknown	unknown	Dynein	[82,140]
			
**Endosomes**			
Rab5	VPS34	KIF16B	[97]
Rab5	HAP40	unkown	[98]
Rab4		KIF3	[99]
Rab4	LIC	Dynein	[100]
Rab7	RILP	Dynein	[101,102]
Rab11	FIP2	Myosin V	[45]
			
**PSD protein**			
PSD-93, PSD-95, S-SCAM,		KIF1Bα	[94]
SAP102, SAP97			
GKAP	DLC	Dynein	[93]
			
**RNA Particle (RNP)**			
unkown		KIF5	[41]
FMRP		KIF5/Dynein	[42]
FMRP		KIF3C	[43]
TLS		Myosin V	[34]

In this review we focus on the basic principles of synaptic cargo transport. We discuss how different motors of the kinesin, dynein and myosin families recognize their cargo and how motor-cargo interactions are regulated. Not covered in this review are detailed descriptions of the different motor protein families [12,18] and specific pathways in neuronal trafficking [14], but we will discuss briefly the current knowledge of general cargo transport mechanisms [10,19,20]. Other excellent reviews cover the role of cytoskeletal organization in cargo trafficking [21,22], receptor trafficking through lateral diffusion in the plasma membrane [23,24], transport mechanisms during neuronal polarization [25] and discuss the relationship between trafficking and neurodegenerative disease [26,27]. We aim to give an overview of the molecular trafficking mechanisms important for the delivery and removal of synaptic proteins. Studying the basic machinery for recognition and transport of synaptic cargos might help us to understand fundamental operational principles of synapse formation, function and plasticity.

### Basic mechanisms regulating motor protein transport

**Figure 1:**
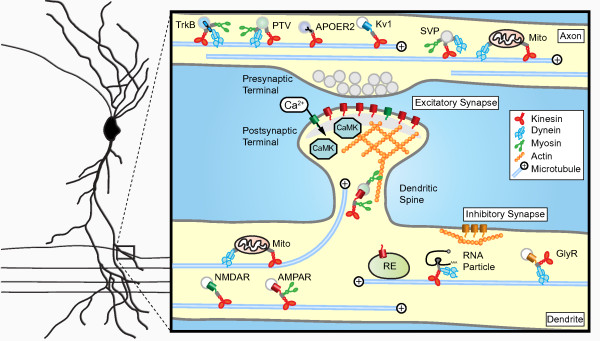
**Basic organization of synaptic cargo transport in the axon and dendrites**. The neuronal plasma membrane features major functional specializations - presynaptic terminals along the axon, which release neurotransmitters, and postsynaptic terminals along the dendrites, which contains neurotransmitter receptors and associated scaffolding proteins. Transport and delivery of synaptic constituents is of key importance for synaptic functioning. Long distance transport through the dendritic or axonal shaft is mainly mediated by microtubule based motors whereas transport over shorter distances is myosin based. Most likely multiple motor proteins, myosin, kinesin and dynein, are bound to the same synaptic cargo. It has recently been shown that dynamic microtubules enter the actin-rich dendritic spine, allowing for both microtubule and actin dependent synaptic cargo transport. Activity regulated transport and targeting of synaptic components has become an attractive model for the regulation of the synapse specific membrane composition. TrkB, tyrosine receptor kinase B; PTV, Piccolo-Bassoon transport vesicles; APOER2, Apolipoprotein E Receptor 2; Kv1, Axonal voltage gated potassium channel; SVP, Synaptic vesicle precursor; Mito, Mitochondria; NMDAR, NMDA receptor; AMPAR, AMPA receptor; RE, Recycling endosome; CaMK, Calcium calmodulin dependent kinase; GlyR, Glycine receptor.

All eukaryotic cells rely on the cytoskeleton to facilitate active transport. For neurons it is especially important to have efficient and well-regulated transport to establish and maintain polarized neuronal morphology and synaptic specializations. Two types of cytoskeletal networks are involved in neuronal transport; the microtubule and actin cytoskeleton. Microtubules are dynamic structures with rapidly growing and shrinking plus-ends and a more stable minus-ends [28]. Microtubules are mainly present in the shafts of the axon and dendrites whereas actin is more abundant in highly dynamic structures such as growth cones and dendritic spines [22,29,30]. In general, cargos are transported over long distances along microtubules before transferring to the actin cytoskeleton for the final part of their journey. A common feature of both actin and microtubule based transport is the fact that force is generated by motor proteins through ATP hydrolysis [12,18,31,32]). The motor proteins that use microtubules as tracks are the minus-end directed dynein and plus-end directed kinesins, whereas myosins move along actin filaments. Neuronal cargo trafficking is achieved by the concerted efforts of both microtubule-based and actin-based motors [8,33-35] (Figure 1). Several classes of myosin motors participate in neural cargo transport in axon and dendrites - most commonly used are myosin V and myosin VI (Table 1). Although the basic mechanisms for microtubule- and actin-dependent transport in neurons are well established, how neuronal cargos achieve specificity and directionality to their site of action is an emerging field of investigation.

#### Microtubule organization in neurons

In neurons, the microtubule cytoskeleton is organized differently in dendrites and axons. Dendritic microtubules have mixed polarity i.e. plus-ends can face either away from or towards the cell body, whereas the microtubules in the axon have a uniform polarity with all plus-ends facing outwards [36,37] (Figure 1). Recently, by imaging microtubules using fluorescently tagged α-tubulin and microtubule plus-end binding proteins it was demonstrated that neuronal microtubules are very dynamic and regularly enter dendritic spines [38,39]. Microtubules grow with their plus-ends directed towards the spine and are able to repeatedly enter the same spine [39] (Figure 1). In contrast, microtubule nucleation within spines and subsequent polymerization into the dendrite shaft has not been observed. Therefore, polarized growth of microtubules into spines could facilitate delivery of synaptic cargo directly to the postsynaptic site (Figure 1). Although microtubule-dependent cargo transport in dendritic spines has not been directly visualized yet, it will be interesting to investigate whether the translocation of excitatory postsynaptic proteins or other cellular components, such as organelles and RNA particles (RNPs), rely on microtubule based transport [40-43] (Table 1). On the other hand, several studies directly demonstrate the importance of the actin-based transport mechanisms at excitatory synapses. Myosin V, for example, mediates RNP translocation into dendritic spines [34], is required for the spine localization of smooth endoplasmic reticulum at Purkinje cell synapses [44] and transports AMPA receptor containing recycling endosomes during synaptic potentiation [45,46].

#### Post-translational modification of microtubules

One way to directly influence synaptic cargo transport is to generate functional diversity by modifying the cytoskeleton. Motor proteins recognize these spatial cues and establish specific synaptic cargo trafficking routes. It has recently been demonstrated that post-translational modification (PTM) of microtubules can alter their stability and motor protein binding characteristics [47]. Microtubules can acquire a variety of evolutionarily conserved PTMs including polyglutamylation, polyglycylation, detyrosination, acetylation, phosphorylation and palmitoylation [48]. In most cases, modifying enzymes act preferentially on tubulin subunits already incorporated into microtubules. It is tempting to speculate that, due to the specific organization of microtubules in neurons, microtubule PTM is of even greater importance than in non-neuronal cells. Recently, it was found that polyglutamylation targets kinesin-3 family member KIF1A motors [49] and tyrosination recruits kinesin-1 family member KIF5 motor proteins [50]. Now, it will be critical to identify the forward and reverse enzymes responsible for the various modifications of neuronal microtubules.

#### Regulation of motor protein activity

The motor domains responsible for motility and force generation are connected by a stalk to the tail domain, which directly or indirectly binds to their cargo [12]. Another possible mode of transport regulation is direct modulation of motor protein activity. Tremendous progress has been made in the mechanistic understanding of molecular motors - both in force generation and directional movement [12,32,51,52]. For instance, a recent study shows that the specific structure of dynein, with six AAA+ ATPase domains, a linker and a microtubule binding stalk, explain the characteristic dynein stepping pattern [53]. The stepping behavior of kinesin was shown to be regulated by intramolecular strain, allowing the kinesin motor to maintain its plus-end directed movement and also coordinate the two motor domains [54]. Moreover, several studies demonstrate that myosin and kinesin motors can fold back on themselves in order to regulate their own motor activity [39]. In the absence of bound cargo, the tail domain interacts with the motor domain and inhibits its activity [55,56]. Furthermore, motor accessory proteins such as dynactin have been shown to increase the processivity of dynein and kinesin-2 [57-59].

#### Regulation of bidirectional transport

Another intriguing aspect of transport regulation lies in the use of multiple motors by a single cargo (Figure 1). Various synaptic cargos were shown to move bidirectionally along the microtubule network and some can even switch between actin and microtubules (Table 1). Several models have been proposed to explain bidirectional transport. The "tug-of-war" and "coordination" models both describe a situation where different types of motors are simultaneously bound to one cargo [20]. In the "tug-of-war" model, motors of both minus-end and plus-end directionality will create a force that is decisive for the direction of cargo transport. The back and forth movement of synaptic cargo can be explained by motor loss or changes in activities of motors, causing the opposing motors to take the lead [60]. The "coordination" model predicts that when one motor is actively engaged on the microtubule, the opposing motor is turned off. Here, disruption of one set of motors causes transport defects in both directions [61]. Both transport machineries are most likely controlled by large regulatory complexes present on the cargo, consisting of Rab GTPases, scaffolding and signaling proteins [10,20]. In this way, signaling proteins can turn one of the motors "on" and the opposing motor "off" and thus coordinate the activity of both motors. It was known from *in vitro *studies that cargos that contain multiple motors were able to move further and faster than cargos which engaged only one motor [62,63]. However, a recent study using *Drosophila *embryos showed that one cargo that engages multiple motors in fact does not travel faster nor does it travel a greater distance [64]. These data suggest that *in vivo *regulation of motor proteins requires additional factors, possibly in the form of motor-adaptor complexes on moving cargo.

### Motor-cargo interactions

**Figure 2:**
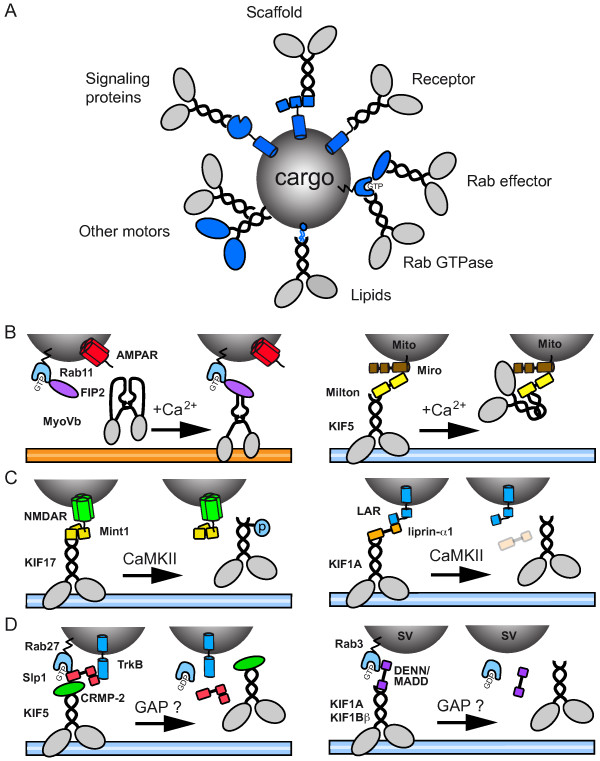
**Regulation of motor-cargo binding**. A) Basic modes of motor-cargo interaction. Several distinct mechanisms are found that dock molecular motors onto cargos - interaction with phospholipids, receptors or integral membrane proteins, other motor proteins, scaffolding proteins, signaling proteins, such as kinases and phosphatases, and small Rab GTPases and their effector proteins. (B-D) Regardless of the cargo or the nature of the motor-cargo interaction, it is clear that a very close connection exists between the regulation of membrane trafficking and the docking of motor proteins to specific cargo. Three recent examples show that tight regulation of the motor-cargo interaction facilitates transport specificity and cargo binding/release at synaptic locations. (B) Ca^2+ ^dependent cargo recruitment and release. Activity stimulated influx of Ca^2+ ^allows unfolding of myosin-V and subsequent binding to the Rab11 effector FIP2 on recycling endosomes containing AMPA receptors. Another example of Ca^2+ ^regulated transport is Miro/Milton dependent transport of mitochondria. Upon binding of Ca^2+^, Miro is able to interact with KIF5's motor domain, thereby preventing microtubule interaction and inhibiting mitochondria transport. (C) CaMKII regulated cargo release. CaMKII dependent phosphorylation of the KIF17 releases NMDA receptor-containing vesicles. In an alternative model active CaMKII is responsible for the degradation of liprin-α1 in turn causing the release of LAR-containing cargo. (D) Rab GTPase dependent transport. Rab27 associates with TrkB vesicles via its effector protein Slp1. The TrkB/Slp1/Rab27 complex binds via CRMP2 to KIF5 and allows transport of TrkB vesicles. Another study shows that Rab3 effector DENN/MADD is an adaptor between KIF1Bβ/KIF1A motors and Rab3-carrying vesicles. Neuronal activity might cause cargo release by activating Rab GTPase-activating proteins (GAPs). NMDAR, NMDA receptor; LAR, leukocyte antigen-related tyrosine phosphatase; AMPAR, AMPA receptor; Mito, mitochondrion; SV, synaptic vesicle; CaMKII, calcium calmodulin dependent kinase; FIP2, Rab11-family interactin protein 2; MyoVb, Myosin Vb; Slp1, synaptotagmin-like 1; TrkB, tyrosine receptor kinase B; CRMP-2, Collapsin response mediator protein 2; DNENN/MADD, differentially expressed in normal versus neoplastic/mitogen-activated protein kinase (MAPK) activating death domain.

Members of all three types of cytoskeletal motors are involved in synaptic cargo trafficking [8,33,35]. To understand cargo transport it is essential to determine how motors and associated proteins link up to distinct cargos and how cargo-specific attachment is coordinated and regulated. The search for cellular components that link motors to cargos yields a wide spectrum of attachment mechanisms (Figure 2A)(Table 1).

#### Motor-lipid binding

The most direct mechanism of membrane association is attachment to the phospholipid bilayer. Myosin I motors that possess a basic tail domain bind to acidic phospholipids [65] and kinesin-3 family members bind to lipids via their pleckstrin homology domain in the tail region [66]. Dynein cannot directly bind to protein-free liposomes, it requires dynactin and spectrin to link to acidic phospholipids in the membrane [59,67].

#### Motor-receptor binding

Motor proteins can interact with receptors or integral membrane proteins on membrane cargo vesicles. Kinectin is a membrane protein in the endoplasmic reticulum and was the first proposed KIF5 receptor to anchor motors on cargo membranes [68,69]. KIF5 has been suggested to bind directly to amyloid precursor protein (APP), a transmembrane protein of which proteolytic fragments give rise to amyloid plaques in Alzheimer's disease patients [70], although some other studies were unable to find evidence for a direct interaction [71]. In photoreceptor cells, the light chain of dynein, Tctex-1, directly binds to the G-protein coupled receptor rhodopsin [72]. Rhodopsin mutations responsible for retinitis pigmentosa inhibit the interaction with dynein. Tctex-1 also interacts with neurotrophin Trk receptors, suggesting that the dynein motor complex is responsible for retrograde transport of Trk receptors [73]. Consistently, real-time imaging revealed that dynein is required for rapid transport of internalized, activated Trk receptors from axon terminals to the cell bodies [74].

#### Motor-motor binding

Synaptic cargos engage multiple types of motors for both actin and microtubule based transport. There is strong evidence that the cooperation of multiple motors on a single cargo is important to regulate cargo transport [19]. Communication between different types of motor on a cargo is most easily envisaged if motors are in direct physical contact. Myosin V and KIF5 have been shown to bind directly and enhance each other's processivity [75,76]. This motor-motor interaction may represent a mechanism by which the transition of vesicles from microtubules to actin filaments is regulated. Moreover, physical interactions between dynein and kinesin motor proteins have also been reported - direct binding between cytoplasmic dynein and KIF5 [77] and Xenopus kinesin-2 associated protein (XKAP) and dynactin has been described [78]. Motor-motor associations may also be a mechanism to coordinate anterograde and retrograde cargo movements along microtubules.

#### Motor-signaling protein binding

Motor proteins can also attach to cargo through binding of signaling proteins and associated components. Jun N-terminal kinase (JNK) interacting proteins (JIPs) are of key importance for axonal cargo trafficking [79] - in *Drosophila*, JIP mutants cause aberrant accumulation of axonal cargos, closely resembling the phenotype of kinesin-1/KIF5 mutants. JIP functions as an adaptor protein by binding directly to kinesin light chain and carries Reelin receptor ApoER2-containing vesicles [80]. Moreover, activation of the JNK signaling pathway triggers cargo release [81].

Both KIF5 and dynein are the primary motors for mitochondria trafficking in *Drosophila *motor axons [82]. The Miro GTPases have recently been demonstrated to anchor in the outer mitochondrial membrane and regulate mitochondrial transport and synaptic function [9]. Mitochondria coated with Miro recruit KIF5 through the association with Milton/TRAK proteins [83]. In flies, loss of Miro and Milton function display similar phenotypes - abnormal clustering of mitochondria in the cell body and impaired synaptic function at the neuromuscular junction [84,85].

#### Motor-scaffold protein binding

Synaptic scaffolds are multifunctional proteins with several protein-protein interaction modules [1,3,7]. Several studies demonstrate that scaffolding proteins bind directly to motors and function as motor-cargo linkers [10,86,87]. The motor-cargo complex on glutamate receptor-containing vesicles consists of glutamate receptor subunits, scaffolding proteins and kinesin motors [88,89]. The NMDA receptor NR2B subunit interacts with KIF17 through a scaffolding protein complex that consists of Lin7 (also known as MALS/Velis), Lin2 (CASK) and Lin10 (Mint1). Consistently, NR2B and KIF17 have been localized on the same vesicle and move within the dendritic shaft [90]. A similar motor-cargo model can be made for AMPA receptor transport - AMPA receptor subunits GluA2/3 (also named GluR2/3) interact with KIF5 through the multiple PDZ-domain containing scaffolding protein GRIP/ABP [88]. Interestingly, GRIP can also bind to KIF1A through GRIP interacting protein liprin-α [91], suggesting that different microtubule-dependent motors are involved in AMPA receptor cargo transport. Moreover, GRIP contains up to seven PDZ domains and might transport many other interacting proteins, such as EphB receptors, their ephrin ligands and transmembrane protein Fraser syndrome 1 (FRAS1). Recently, GRIP has been implicated in dendrite morphogenesis by serving as an adaptor protein for EphB receptors [92]. Synaptic scaffolding protein GKAP interacts with dynein light chain [93] and PSD-95 and related PDZ domain containing scaffolding proteins associate with KIF1Bα, however their function as a motor-cargo adaptors has not been shown directly [94] (Table 1). However, PDZ domain containing protein SAP102 has been implicated in NMDA receptor trafficking [95].

#### Motor-Rab GTPase binding

Motor proteins also make use of small Rab GTPases, often with the help of Rab effector molecules, to attach to their cargo. Several secretory and endosomal Rab proteins have been shown to bind to specific motors [96-103] (Table 1). Recently, it was shown that Rab3 effector DENN/MADD is an important linker between KIF1Bβ/KIF1A motors and Rab3-containing synaptic vesicle precursors [104]. Surprisingly, the number of synaptic vesicles is not reduced in quadruple Rab3A-D knockout mice [105], suggesting that redundant synaptic vesicle trafficking mechanisms are present. Recently, Rab6 was shown to regulate transport and targeting of secretory vesicles [106]. Rab6 binds the dynein/dynactin motor complex through its effector protein Bicaudal-D (BICD) and regulates microtubule-minus end directed transport [92,107,108]. Moreover, recycling endosome GTPase Rab11 was shown to interact with myosin Vb and regulate AMPA receptor trafficking [45,109]. Myosin Vb binds to Rab11 through its effector Rab11-family interacting protein 2 (Rab11-FIP2) allowing the transport of AMPAR containing recycling endosomes into the dendritic spine [45].

### Synaptic cargo trafficking regulated at the level of motor-cargo binding

Regulation of membrane trafficking is tightly correlated with the docking of motor proteins to specific cargo. It is crucial to understand how cargo trafficking is regulated at early transport events, during motor-cargo binding and motor activation and late transport events including cargo release and retention at the final destination. Several signaling pathways have been found that influence synaptic cargo movement and distribution, such as phosphorylation-dependent signaling [110-113]. Here we focus on the recent studies demonstrating how motor-cargo interactions are regulated by neuronal activity.

#### Ca^2+ ^levels regulate motor-cargo binding

One way to control intracellular trafficking is to regulate motor-cargo interactions by responding to changes in local ionic concentrations. Activation of NMDA receptors causes a rapid influx of Ca^2+ ^in dendritic spines [114]. A recent study shows that Myosin Vb is a "Ca^2+ ^sensor" for actin-based postsynaptic AMPA receptor trafficking [45]. Increased Ca^2+ ^levels lead to unfolding of Myosin Vb motors and allows for binding to Rab11-FIP2 adaptors on recycling endosomes [115,116] (Figure 2B). The association of myosin Vb with Rab11-FIP2 recruits AMPA receptor containing recycling endosomes into spines. Thus, elevated Ca^2+ ^levels in spines promote actin-based postsynaptic trafficking. On the other hand, Ca^2+ ^influx reduces mitochondrial motility [117]. Recent studies suggest that the mitochondrial Miro-Milton adaptor complex is essential for the Ca^2+^-dependent regulation of mitochondria trafficking [118]. Elevated Ca^2+ ^levels permit Miro to interact directly with the motor domain of KIF5 (Figure 2B). The interesting aspect of this model is that KIF5 remains associated with mitochondria regardless of whether they are moving or stationary. In the "moving" state, KIF5 is bound to mitochondria by binding to Milton, which in turn interacts with Miro on the mitochondrial surface. In the "stationary" state, in presence of high Ca^2+ ^levels, the KIF5 motor domain interacts directly with Miro and prevents microtubule interactions. Both findings imply the existence of "Ca^2+ ^sensors" that detect neuronal activity stimuli and convert Ca^2+ ^influx into mechanisms regulating cargo trafficking.

#### CaMKII signaling regulates motor-cargo binding

Ca^2+ ^influx also triggers synaptic signaling cascades [119,120]. A key player in this process is calcium/calmodulin-dependent protein kinase II (CaMKII), a calcium-activated serine/threonine kinase that is present at synaptic terminals. Recent findings link CaMKII activity with regulated motor-cargo binding. For instance, CaMKII has been shown to phosphorylate KIF17, which induces the dissociation of the Mint1 scaffolding protein complex and release of NMDA receptor-containing cargo near the postsynaptic membrane [90] (Figure 2C). In this way, regulated CaMKII activity provides an attractive mechanism for targeting NMDA receptor complexes to active synapses where the kinase is switched "on". CaMKII has also been shown to modulate myosin V - cargo binding in *Xenopus *melanophores [121]. It will be interesting to test whether myosin V is important for NMDA receptor-containing vesicle transport. Another recent study shows that CaMKII controls the trafficking of leukocyte common antigen-related (LAR) family of receptor protein tyrosine phosphatases (Figure 2C) [122]. LAR associates with KIF1A via the adaptor protein liprin-α1 [91,123,124]. Interestingly, liprin-α1 is degraded in response to CaMKII phosphorylation and additionally regulated by the ubiquitin-proteasome system [122]. At synapses where CAMKII is active, LAR-liprin-α1 containing vesicles would be "unloaded" due to CaMKII-mediated degradation of liprin-α1. An attractive idea is that the regulated degradation of cargo-adaptors or motor proteins is a general molecular mechanism for directed motor trafficking.

#### Rab GTPases regulate motor-cargo binding

Rab proteins are molecular switches which cycle between a GDP-bound and a GTP-bound state and represent the inactive and active states, respectively [125]. Activation of Rab GTPases is mediated by predominantly membrane-associated Rab guanine nucleotide exchange factor GEFs, inactivation of Rabs is a consequence of their intrinsic capacity to hydrolyse GTP. However, this activity is low and requires stimulation by Rab GTPase-activating proteins (GAPs). An additional level of regulation exists through guanine nucleotide dissociation inhibitors (GDIs), which prevent activation of GTPases by blocking their interaction with GEFs and effector proteins. Recent studies show that motor-cargo interactions are controlled by regulated Rab GTPase activity.

Collapsin response mediator protein-2 (CRMP-2) is crucial for axon formation in hippocampal neurons and functions as a motor-adaptor protein to link KIF5 to neurotrophin Trk receptors [126]. CRMP-2 forms a complex with Rab27 and its effector Slp1, associates with the cytoplasmic region of TrkB and regulates anterograde transport of TrkB [127]. Interestingly, the direct binding of TrkB to Slp1 is dependent on the GTPase activity of Rab27. The binding between Slp1 and GTP-bound Rab27 might be regulated by a Rab27 GAP mediated block of Rab inactivation (Figure 2D). On the other hand it is possible that Rab27 specific GDIs and GEFs regulate the recruitment to TrkB-containing vesicles. Another study shows that the nucleotide-bound Rab3 regulates trafficking of KIF1Bβ/KIF1A-positive synaptic vesicle precursors through the interaction with its effector protein DENN/MADD [104]. GTP-Rab3 is more effectively transported than GDP-Rab3 in axons and Rab3 activity can regulate the interaction between KIF1Bβ/KIF1A-DENN/MADD and Rab3-containing synaptic vesicle precursors (Figure 2D). It will be interesting to find signaling pathways leading to the regulation of Rab3 GTPase activity. Interestingly, DENN/MADD has been shown to function as a GEF for Rab3 [128]. Identifying GEFs and GAPs for specific Rabs will be a major future challenge to understand how Rab GTPases regulate motor-cargo binding and transport specificity.

## Concluding remarks and future perspectives

During the last decade a number of factors have been identified that interact with specific motor proteins. Biochemical and proteomics approaches and high-throughput yeast two-hybrid screens have identified more than 100 proteins that bind to kinesin-1/KIF5 in mammals, flies and worms [129]. Most of these proteins act as cargo molecules themselves, function as motor-adaptor proteins (scaffolding proteins, Rab GTPases, signaling proteins) or are regulators of motor activity. It is becoming increasingly clear that motor-adaptor-cargo interactions play a key role in identifying synaptic cargos and regulating synaptic cargo trafficking. As such, these complexes function as important regulators of synaptic structure and function.

Synaptic cargos often move bidirectionally in neurons, employing both plus-end and minus-end directed motors along microtubules, and can even switch between actin and microtubule tracks. It is very likely that transport of cargo to synaptic sites is controlled by the cooperation of several types of motor proteins for both actin and microtubule based transport. For example, multiple motor proteins contribute to the transport of AMPA receptors, each binding directly or indirectly to the AMPA receptor protein complex (Table 1) - GluA1 binds to myosin V, GluA1 interacts with myosin VI (via SAP-97), GluA2 binds to KIF5 (via GRIP) and GluA2 interacts with KIF1A (via GRIP-liprin-α) [46,88,91,130]. The same holds true for other receptors, such as glycine receptors [131,132] and Trk receptors [73,127,133], synaptic vesicle precursors [66,91,104,134], piccolo-bassoon transport vesicles [135-137] and mitochondria [82,83,138-140] (Table 1). If these motors are all attached to a single synaptic cargo simultaneously, transport can easily be adjusted or even reversed by regulating the activity of the different motors. This mechanism is likely to be much quicker than assembling a new set of motors on a moving synaptic cargo, and also allows synaptic transport to be quickly altered depending on local ion concentrations and other synaptic signaling mechanisms. It even makes it possible to precisely tune transport in such a way that synaptic cargos can seamlessly switch from microtubules to actin. This might be crucial for positioning synaptic cargos at their specific place of action - microtubule dependent motors deliver synaptic cargo to the entrance of synaptic sites and myosin motors facilitate the transport into synapses. Interestingly, by imaging super-ecliptic pHluorin-tagged AMPA receptors, GluA1 insertions were seen at the extrasynaptic surfaces [141,142]. If so, synaptic delivery of AMPA receptors may require an additional trafficking step involving lateral diffusion from extrasynaptic pools to synaptic sites [24]. Similar three-step mechanisms may be responsible for the delivery of other synaptic components at both pre- and postsynaptic sites. The cooperation between different motor proteins in synaptic cargo transport raises entirely new questions about motor protein regulation and synaptic cargo docking. Identification of new motor-adaptor-cargo regulators will increase our understanding of transport regulation. Since adaptor proteins play a crucial role in the transport of synaptic cargo, they can serve as excellent starting points to identify other components of the machinery.

One major challenge is to visualize synaptic cargo transport along single microtubule tracks. This is important since dendrites do not have a uniform microtubule orientation and many microtubules are aligned anti-parallel to each other (Figure 1). In this way, minus- and plus-end directed motors move towards both ends of a dendrite. As a consequence, visualizing synaptic cargo trafficking alone does not identify the motor driving cargo motility. The unique microtubule organization in dendrites raises additional questions about the coordination of directed postsynaptic cargo trafficking. To better understand cargo trafficking in dendrites, more knowledge is needed on dendritic microtubule organization as well as novel approaches to visualize synaptic cargo transport. Recent studies started to address the role of dynamic microtubule organization in dendrites [39], investigate the influence of neuronal activity on microtubule PTM [132] and study the effect of long-term microtubule stabilization on neuronal polarity [143]. The spatial and temporal arrangement of transport complexes and the underlying cytoskeleton is likely to be important to understand how multiple motors can regulate specific synaptic cargo transport and delivery.

## Competing interests

The authors declare that they have no competing interests.

## Authors' contributions

Both authors participated in developing the ideas, the writing, discussion and integration of the information. Both authors read and approved the final manuscript.

## Authors' information

M.A.S is a PhD student in the lab of C.C.H. He graduated with a B.Sc and M.Sc in Biology from Leiden University, The Netherlands, and a M.Sc in Neuroscience from Erasmus University Rotterdam, The Netherlands. C.C.H is an Associate Professor at the Erasmus Medical Center in Department of Neuroscience. He has been a postdoctoral fellow in the lab of Morgan Sheng at the Picower Center for Learning and Memory at the Massachusetts Institute of Technology, USA. He graduated with a B.Sc in Biochemistry from the Hogeschool Rotterdam in The Netherlands, followed by a M.Sc in Biology from Utrecht University, The Netherlands, and a Ph.D in Molecular and Cellular Biology from Erasmus University, Rotterdam, The Netherlands.
